# Multiomics integration-based molecular characterizations of COVID-19

**DOI:** 10.1093/bib/bbab485

**Published:** 2021-12-02

**Authors:** Chuan-Xing Li, Jing Gao, Zicheng Zhang, Lu Chen, Xun Li, Meng Zhou, Åsa M Wheelock

**Affiliations:** Respiratory Medicine Unit, Department of Medicine & Centre for Molecular Medicine, Karolinska Institutet, Stockholm, Sweden; The First Hospital of Lanzhou University, Lanzhou, China; Respiratory Medicine Unit, Department of Medicine & Centre for Molecular Medicine, Karolinska Institutet, Stockholm, Sweden; Heart and Lung Centre, Department of Pulmonary Medicine, University of Helsinki and Helsinki University Hospital, Helsinki, Finland; The First School of Clinical Medicine, Lanzhou University, Lanzhou, China; School of Biomedical Engineering, School of Ophthalmology & Optometry and Eye Hospital, Wenzhou Medical University, Wenzhou, China; School of Biomedical Engineering, School of Ophthalmology & Optometry and Eye Hospital, Wenzhou Medical University, Wenzhou, China; The First Hospital of Lanzhou University, Lanzhou, China; The First School of Clinical Medicine, Lanzhou University, Lanzhou, China; Department of General Surgery, The First Hospital of Lanzhou University, Lanzhou, China; Key Laboratory of Biotherapy and Regenerative Medicine of Gansu Province, The First Hospital of Lanzhou University, Lanzhou, China; School of Biomedical Engineering, School of Ophthalmology & Optometry and Eye Hospital, Wenzhou Medical University, Wenzhou, China; Respiratory Medicine Unit, Department of Medicine & Centre for Molecular Medicine, Karolinska Institutet, Stockholm, Sweden

**Keywords:** COVID-19, multiomics integration, molecular characteristics, severity, outcome, single-cell omics

## Abstract

The coronavirus disease 2019 (COVID-19) pandemic, caused by the severe acute respiratory syndrome coronavirus 2 (SARS-CoV-2), rapidly became a global health challenge, leading to unprecedented social and economic consequences. The mechanisms behind the pathogenesis of SARS-CoV-2 are both unique and complex. Omics-scale studies are emerging rapidly and offer a tremendous potential to unravel the puzzle of SARS-CoV-2 pathobiology, as well as moving forward with diagnostics, potential drug targets, risk stratification, therapeutic responses, vaccine development and therapeutic innovation. This review summarizes various aspects of understanding multiomics integration-based molecular characterizations of COVID-19, which to date include the integration of transcriptomics, proteomics, genomics, lipidomics, immunomics and metabolomics to explore virus targets and developing suitable therapeutic solutions through systems biology tools. Furthermore, this review also covers an abridgment of omics investigations related to disease pathogenesis and virulence, the role of host genetic variation and a broad array of immune and inflammatory phenotypes contributing to understanding COVID-19 traits. Insights into this review, which combines existing strategies and multiomics integration profiling, may help further advance our knowledge of COVID-19.

## Introduction

Coronavirus disease 2019 (COVID-19) was first detected in China in December 2019, and was subsequently declared a global pandemic by the World Health Organization in March 2020 [1–3]. Severe acute respiratory syndrome (SARS) coronavirus 2 (SARS-CoV-2) is the etiological driver of COVID-19 [4], representing a major global health challenge that follows outbreaks of the Middle East respiratory syndrome CoV (MERS-CoV) and severe acute respiratory syndrome CoV (SARS-CoV) [2, 3, 5]. SARS-CoV-2 is a positive-sense single-stranded RNA virus with enveloped virions, which primarily target the epithelial cells, typically causing respiratory tract illnesses potentially leading to severe pneumonia and acute respiratory distress syndrome (ARDS) [2, 6]. As of September 2021, more than 232 million COVID-19 cases and over 4 million associated fatalities have been reported affecting 220 countries and territories [7]. The entire world continues to battle the wave of COVID-19, although extraordinary efforts to prevent and treat the disease have been carried out globally to date, including lockdowns of cities and countries, as well as the widespread rollout of vaccines, supportive therapy, and treatment to prevent respiratory failure [8, 9]. SARS-CoV-2-induced COVID-19 in many ways resembles SARS caused by SARS-CoV given the acute clinical presentation, although with the addition of previously unknown peculiar pathogenetic, epidemiological and clinical features, thereby adding to the complexity of COVID-19 pathogenesis [3]. The estimated COVID-19 mortality is approximately 2.3%, lower than that for SARS (9.5%) and MERS (34.4%) [3], with the risk of COVID-19 mortality increasing with age and comorbidities, such that more than 20% of all reported disease-associated deaths have occurred in nursing homes in some countries [10]. COVID-19 has been shown to spread more readily in the community than MERS and SARS, primarily due to three major factors: (i) a large proportion of SARS-CoV-2 infected people display mild-to-no COVID-19 clinical symptoms [11]; (ii) SARS-CoV-2 appears to be contagious prior to symptom onset [12]; (iii) SARS-CoV-2 appears to primarily be transmitted through aerosols from exhaled air, which can linger in poorly ventilated areas for several hours, thereby contributing to so-called super-spreader events [13]. Further considering inconsistencies in the reporting of data from varying sources, tracing and estimating the spread of the pandemic remains challenging [10]. The manifestations of COVID-19 exhibit a range of differences from asymptomatic to exceptionally severe, with the possibility of presenting multiple systemic symptoms, including common symptoms such as a fever, shortness of breath, a persistent dry cough, chills, muscle pain, headache, a loss of taste or smell, renal dysfunction and gastrointestinal symptoms [3, 14, 15]. Age- and sex-related differences in both presentation and severity of COVID-19 disease are well documented [10, 16]. Multi-organ damage in COVID-19 patients has also been reported [1]. Furthermore, elderly COVID-19 patients with other comorbidities may present with severe respiratory manifestations [17]. Hence, an increased and comprehensive understanding of COVID-19 characteristics is crucial to guiding the pandemic response [10], and integrating multiple data modalities from the same patients in combination with appropriate patient stratification may prove advantageous in understanding COVID-19 disease.

Despite the rapid global scientific response to COVID-19, disease management and scientific research remain emergency-level challenges. Evidence indicates that both viral and host factors play critical roles in COVID-19, with host factors closely impacting disease severity [18, 19]. Multiomics datasets of high-throughput data, protein interactions and functional annotation profiling and approaches may provide a comprehensive understanding of the heterogeneity and diversity of data types and complex internal relationships in COVID-19. Identifying these characteristics using multiomics integration profiling covering specific stages and severities of COVID-19 disease may lead to a better understanding of specific virus variant characteristics and individual responses, disease manifestations and therapeutic opportunities, all of which facilitate the accurate prediction of patient outcomes as well as prevention and intervention in a personalized medicine manner. Moreover, novel multiomics approaches could contribute to increased statistical power in small cohorts [20] and reduce the risk of overfitting statistical analyses. By summarizing the multiomics integration profiles of COVID-19, we may identify preventive and therapeutic targets along with early diagnostic tools, and more effectively predict critical or severe outcomes, better select drugs, and further our understanding of comorbid conditions and the long-term complications associated with COVID-19. In this review, we summarize the multiomics integration data published to date, including the integration of proteome, metabolome, lipidome, transcriptome, interactome, genome, secretome and cytokine, immune-related signatures and drug omics data to characterize a range of acute to long-term manifestations of COVID-19. We first summarize the applications of multiomics-based molecular characterizations in COVID-19 based on clinical stages and questions, and then, we group the major bioinformatics approaches used in multiomics integration analyses of COVID-19. Finally, we discuss various trends and challenges, such as the rapid growth of single-cell omics and network-based approaches. We argue that the use of multimodality integration situates COVID-19 within the purview of precision medicine, potentially improving disease management in the future.

## Application of multiomics-based molecular characterization to COVID-19

**Figure 1:**
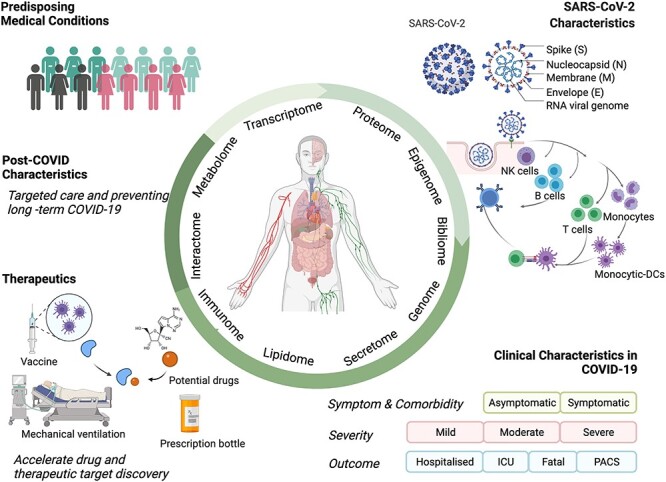
Applications of the multiomics integration-based molecular characterization of COVID-19. COVID-19, coronavirus disease 2019; SARS-CoV-2, severe acute respiratory syndrome coronavirus 2; MERS-CoV, Middle East respiratory syndrome CoV; ICU, intensive care unit; PACS, post-acute COVID syndrome. Created using BioRender.com.

A striking feature of COVID-19 is its heterogeneity [21], involving diverse underlying pathophysiological processes [22]. Clinical manifestations differ with age, although populations of all ages are susceptible to SARS-CoV-2 infection [17]. For instance risk factors for developing severe respiratory disease include higher age, male sex, and a number of pre-existing chronic disease, particularly multi-morbidity [17]; female gender is a risk factor for the long-term symptoms of fatigue combined with vagual and/or respiratory dysfunction associated with post-acute COVID syndrome (PACS), whereas the majority of previously healthy young adults, youth and children present with mild or even asymptomatic disease [23, 24]. Multiple symptoms have been reported in COVID-19, including but not limited to fever, cough, myalgia, hemoptysis, diarrhea and olfactory and taste disorders [25–29]. Multiomics data integration analyses could contribute to a better understanding of the molecular intricacy and variations of disease, providing new opportunities for studying COVID-19 in a more comprehensive manner given the unprecedented dimensionality and diversity of data available [30]. In this review, we highlight investigations of COVID-19 combining samples from multiple anatomical compartments, such as blood, bronchoalveolar lavage fluid (BALF), cell lines, throat swabs and tissues, combined with analyses from multiple molecular levels, such as DNA, mRNA, miRNA, proteins and metabolites. In addition, we provide an overview of how multiomics integration techniques contributed to the discovery of the molecular characteristics of COVID-19, including the virus and its functions, in addition to disease manifestations, severity and progression. Specifically, we summarize how multiple omics data can be applied to address issues such as predicting patient outcomes, identifying novel molecular targets for therapeutic interventions, and providing insights into data on COVID-19 [30] (Figure 1 and Table 1).

**Table 1:** Summary of the main biospecimen type and multiple omics data blocks from COVID-19 multiomics publications

**Authors**	**Main biospecimen types**	**Proteome**	**Metabolome**	**Lipidome**	**Transcriptome**	**Interactome**	**Genome**	**Secretome/Cytokine**	**Immunome/Signature**	**Other omics**
Su et al. [37]	Plasma	bulk	bulk							
	PBMCs	sc						sc	TCR and BCR	
Overmyer et al. [18]	Plasma	bulk	bulk	bulk						
	Leukocyte				bulk					
Chen et al. [1]	Plasma	bulk	bulk		exRNA					
	Blood sample				bulk					
Thomas et al. [14]	Red blood cells	bulk	bulk	bulk						
Zhou et al. [54]	Bronchial epithelial cells				bulk and sc					
	Cell line (Caco-2 cells)	bulk								
	Unspecified					PPI				
Tomazou et al. [57]	Serum	bulk	bulk							
	BALF				bulk					
	PBMCs				bulk					
	Cell lines (A549, Calu-3 and NHBE)				bulk					
	Lung sample				bulk					
	Unspecified					PPI	GWAs			
Barh et al. [21] Barh et al. [38] Barh et al. [46]	Cell line (Caco-2 cells)	bulk								
	BALF				bulk					
	PBMCs				bulk					
	Cell lines^a^				bulk					
	Unspecified					PPI				Bibliome
Zhao et al. [73]	Colostrum	bulk	bulk	bulk						
Yang et al. [59]^*^	Unspecified		bulk		bulk					
Gupta et al. [32]	SARS-CoV-2 viruses						bulk			
	Unspecified					PPI				
Shen et al. [70]	Serum	bulk	bulk							
Song et al. [69]	Plasma		bulk	bulk						
Young et al. [33]	Plasma						bulk (∆382)		immune mediators	
	Respiratory samples						bulk (∆382)			
Bruzzone et al. [39]	Serum		bulk	bulk						
Barberis et al. [78]	Plasma		bulk	bulk						
Islam et al. [35]	Nasopharyngeal samples				bulk		bulk			
	Lung sample				bulk					
	Cell lines (Calu-3 cells and NHBE)				bulk					
	Unspecified					PPI				
Chen et al. [58]	Plasma	bulk	bulk	bulk						
	Leukocyte				bulk					
	Cell line (Caco-2 cells)	bulk			bulk					
Sciacchitano et al. [55]	PBMCs						bulk	cytokine	immunological profiles	
Muthuramalingam et al. [60]	Blood sample				bulk				immuno-transcriptome	
	Unspecified					PPI				
Bernardes et al. [49]	Blood sample				bulk				bulk BCR-seq	Epigenome
	PBMCs				sc				scBCR-seq	
	Serum							cytokine	antiviral antibodies	
Sun et al. [36]	Serum	bulk	bulk	bulk	bulk					
Terracciano et al. [71]	Unspecified					PPI				
	PBMCs	bulk								
Stukalov et al. [40]	Cell line (A549 cells)	bulk			bulk					Ubiquitinome; Phosphoproeome
	Unspecified					PPI				
Chen et al. [79]^**^	Lung sample	bulk			bulk				immune cells	
Mcreynolds et al. [50]	Plasma			Bulk				Cytokine		
Chen et al. [80]	Serum	Bulk								
Ahmed et al. [41]	Cell line (NHBE)				Bulk					
	Unspecified	Bulk				PPI; miRomics				Bibliome
Galbraith et al. [22]	Plasma	Bulk	Bulk					Bulk	Seroconversion	
	PBMCs								immune mapping	
	Blood sample				Bulk					
	Red blood cells		Bulk							
Stephenson et al. [47]	PBMCs	sc			sc				TCR and BCR	
Liu et al. [56]	PBMCs	sc			sc				TCR and BCR	
	Blood sample	Bulk						Bulk		

Note: Unspecified biospecimen types represent data resources from multiple or unspecified samples such as PPI or literature (bibliome). PBMCs, peripheral blood mononuclear cells; sc, single-cell; TCR, T-cell receptor; BCR, B-cell receptor; BALF, bronchoalveolar lavage fluid; PPI, protein–protein interaction; NHBE, normal human bronchial epithelial cells; GWAs, association results from the genome-wide association study； ∆382, 382-nucleotide deletion; exRNA, extracellular RNA; Cell lines^a^, human lung epithelium-derived cell lines.

^*^Performed metabolomic profiling of Qingfei Paidu Decoction and transcriptomic profiling of a pneumonia rat model [81].

^**^Multiple proteome data blocks from several longitudinal timepoints. The full list and descriptions for all these publications including the purposes and summary of results, number of samples or cell lines are provided in Supplementary Table 1. The online searchable format shiny app can be found at ‘Collection of multiomics datasets in COVID-19’ (https://zhougroup.shinyapps.io/moCOVID/).

### Multiomics investigation of SARS-CoV-2 characteristics and host responses

An integrated approach to investigations of SARS-CoV-2 could facilitate the study of complex biological processes holistically, while the integration of multiomics data highlights the interrelationships amongst the involved biomolecules and their functions [30]. As an unbiased data-driven technique [31], high-throughput omics can explore the complex characteristics of SARS-CoV-2 from multiple levels. Hence, multiomics and subsequent integrated analyses provide an opportunity to better understand SARS-CoV-2, facilitating insights into the pathophysiological heterogeneity of COVID-19 patients. Here, we summarize several examples to further explain how a multiomics approach helped advance knowledge of SARS-CoV-2. In this review, we summarize methods and results from various studies through an integrative omics approach, clarifying their ability to address applications in SARS-CoV-2, such as examining mutations, host responses, infection biology, transmission characteristics and candidate drugs.

#### To explore SARS-CoV-2 mutation and host responses

Identifying the characteristics of COVID-19 strains helps better understand the virus’ successful invasion and transmission, potentially aiding an understanding of the population-specific variations leading to a high rate of SARS-CoV-2 infections [32]. For example, Gupta et al. [32] presented a global view of the mutational pattern of SARS-CoV-2 through a multiomics levels (genome, proteome and interactome) using comparative genomics and integrated network approach, unveiling the phylogenic patterns, co-mutational hot spots, functional cross-talk and regulatory interactions in SARS-CoV-2. Coronaviruses generally mutate to a limited degree compared to other types of viruses due to the presence of a high fidelity proofreading function during RNA replication. However, the high case load worldwide has resulted in an accumulation of single nucleotide polymorphisms (SNPs) resulting in the occurrence of a number of new variants [32]. The comparative genomics and integrated network study presented five central clades (a, b, c, d and e (e1 and e2)), which were identified and distinguished through co-mutational combinations in COVID-19. This includes clades d and e2, found only in the US strains [32]. The 382-nucleotide deletion (∆382) variant of SARS-CoV-2 was presented to the world, and an observational study using multiomics data clarified the association between the ∆382 variant of SARS-CoV-2 and mild infection [33]. Moreover, van Dorp et al. [34] reported mutations to SARS-CoV-2 at both the genomic and protein levels. Yet, a multiomics integration study showed that around 67% of SNP mutations occurred at the amino acid level in COVID-19, although the distribution of SNP mutations and amino acid variations (AAVs) in the virus were not uniform [32]. In addition, the proteomic and genomic study identified critical proteins in COVID-19, whilst high-frequency AAV mutations were present in these proteins [32]. A mutation in SARS-CoV-2 proteins inhibits viral penetration to the host, and multiomics integration studies describe the viral behavior, the host response, and the virus–host interaction following SARS-CoV-2 infection [32, 35, 36]. Transcriptomes and a comparative study revealed disparate host responses against SARS-CoV-2, wherein innate immunity, interferon and cytokine stimulation served as key factors in the host-induced response to COVID-19 [35]. Multiomics results demonstrated that MYO-5 (A, B and C) proteins served as key host partners in COVID-19, strongly interacting with viral proteins (N, S and M) [32]. The viral proteins play critical roles in conferring SARS-CoV-2 pathogenicity, such as the presence of CpG dinucleotides in N and Nsp1 proteins, which may be involved in regulating pathogenesis [32]. Furthermore, virus–host protein interactions play a critical role in regulating the viral life cycle in COVID-19, where the generation of protein–protein interactions revealed that SARS-CoV-2 hijacked and/or altered cellular processes [36]. Comparative genomics and an integrated network established a host–pathogen interaction (HPI) model, which could serve as the fundamental basis for the structure-guided pathogenesis process inside the host cell in COVID-19 [32]. An multiomics integration approach described the observed SARS-CoV-2 virus characteristics and transmission globally, contributing to the development of tailor-made strategies to COVID-19 [32, 33, 35–37].

#### To understand SARS-CoV-2 biology and similarities

Compared to single omics research in COVID-19, integrated proteome, transcriptome, interactome and bibliome data from COVID-19 research details the biological factors associated with SARS-CoV-2 infection, including more enriched processes such as neutrophil degranulation in the virus [38]. For instance, Barh et al. [38] integrated multiomics (interactome, proteome, transcriptome and bibliome profiles) with host genetic information, ultimately identifying the SAR S-CoV-2 infection biology, potential drugs and prophylaxis agents against this virus. Furthermore, that study demonstrated that the primary interactions of the virus are related to the innate immune pathways, the host translation machinery and the Cullin ubiquitin ligase complex, whereas the critical pathway in SARS-CoV-2 infection is associated with the virus process, mRNA splicing, cytokine and interferon signaling, and ubiquitin-mediated proteolysis [38]. Dysregulated metabolomic and lipidomic profiles in SARS-CoV-2 infection revealed that the pathogenic redistribution of the lipoprotein is related to a high risk of atherosclerosis, whilst increased levels of ketone bodies associate with liver damage [39]. Furthermore, a multiomics integration (transcriptome, proteome, ubiquitinome and phosphoproteome) study of SARS-CoV-2 and SARS-CoV identified both distinct and shared molecular mechanisms in these related coronaviruses [40]. Notably, the two viruses exhibited different abilities to modulate both mitochondrial function and homeostasis through nonstructural protein 2 (NSP2), whilst dysregulating the TGF-β pathway and autophagy in SARS-CoV-2 respectively refer to the open reading frame 8 (ORF8) and ORF3, indicative of the important characteristics of SARS-CoV-2 biology [40]. A lung-specific protein interactomes study identified the hub proteins based on the expression data of SARS-CoV-2 infections, and the hub proteins are merely associated with MERS and human coronaviruses [41]. In addition, the multiomics-based identification of SARS-CoV-2 infection demonstrated that the virus shared a pathway with other diseases, including other virus-related diseases (i.e. influenza A, hepatitis virus, human T-lymphotropic virus 1, the Epstein–Barr virus and measles) as well as non-virus-related diseases (i.e. protozoan, bacterial and parasitic) [38]. By clarifying the biology and similarities of SARS-CoV-2, such findings could not only promote drugs development to block multiple infection pathways, but also guide virus- and host-dependent therapies in COVID-19 [40].

### Identification of disease manifestations

#### To distinguish symptoms of COVID-19

The clinical presentation of COVID-19 consists of asymptomatic and symptomatic patients, but asymptomatic patients present with positive detections of the nucleic acid of SARS-CoV-2 and can transmit the virus to others [11]. A previous study summarized the most common short-term clinical symptoms of COVID-19, which included fever, cough, fatigue, dyspnea, sputum, diarrhea, nasal congestion and emotional disturbances [42–44]. Multiple pathophysiological processes have been implicated in the etiology of severe COVID-19 symptoms, for which integrated omics data could elucidate the interplay between these mutual processes [36, 45]. Barh et al. [46] identified mild and severe symptoms, associated comorbid conditions, and short- and long-term complications of COVID-19, using a multiomics (proteome, transcriptome, interactome and bibliome) based bioinformatics approach, an approach with a precision exceeding 90%. In the multiomics-associated prediction model, 36 viral, 53 short-term, 62 short- to mid-long-term, 194 mid-long-term and 57 congenital conditions were identified amongst specific conditions [46]. Using this model, an average of 93% enriched conditions were identified as associated with COVID-19 [46]. Notably, a dry cough and loss of taste were excluded, although a cough has been reported as a common clinical symptom of COVID-19, highlighting the importance of the comprehensive knowledge needed to capture the clinical picture of COVID-19 [46]. In addition, a single-cell multiomics study presented the symptom-related mechanics of COVID-19, specifically identifying the relative loss of IgA2 in symptomatic disease [47]. These multiomics-based results describe symptom-related risk factors in individuals, thereby helping to fill current gaps in personalized approaches to COVID-19.

#### To determine severity in COVID-19

The clinical course of COVID-19 infection is highly variable in severity, initially falling into four types: mild, moderate, severe and critical [11]. Multiple symptoms accompany COVID-19, ranging from mild to critical [11, 48]. Patients with pneumonia show abnormal chest CT findings, with severe patients frequently requiring artificial ventilation [27, 48]. Multiomics research could provide better insight into the molecular features with cross-ome correlations further explaining any observed differences [18]. Quantified transcriptomic, proteomic, metabolic and lipidemic profiles uncovered correlations between various biomolecule classes, and allowed for the further mapping of critical molecular features of COVID-19, particularly in terms of status and severity [18]. The features were closely correlated to complementary activation, neutrophil activation and dysregulated lipid transport in COVID-19 [18]. Anticoagulation strategies remain contraindicated in COVID-19 with varying severities, whereas an integrated omics study provided novel insights into the field and identified a COVID-19 phenotype characterized by hypercoagulation [18]. Su et al. [37] reported a sharp disease-state shift between mild and moderate COVID-19 using integrated bulk and single-cell multiomics data, whereby therapeutic interventions are likely to be most effective for moderate cases, including an increase in inflammatory signaling and a loss of some sets of metabolites and metabolic processes. Furthermore, multiple unusual immune cell phenotypes emerged in moderate COVID-19 and amplified with increasing disease severity [37]. Interestingly, these novel subsets did not appear with mild COVID-19, whilst sharp differences emerged when comparing mild and moderate disease, involving inflammation signals, immune cell function and plasma metabolite composition [37]. Another multiomics dataset showed that tissue-specific proteins and extracellular RNA (exRNA) expression significantly differed between mild and severe COVID-19 patients [1]. Proteomic, metabolic, transcriptomic and cytokine data identified milder disease symptoms, which accompanied significant T-cell responses [1], whilst single-cell multiomics data revealed that circulating follicular helper T-cells accompanied mild COVID-19 [47]. Moreover, a single-cell multiomics study also demonstrated an increased level of T-cells in severe COVID-19, including an increased ratio of CD8+ effector T-cells to effector memory T-cells and expanded CD8+ T-cells [47]. Nevertheless, several biomarkers were identified in severe COVID-19 based on multiomics studies, such as megakaryocytes, erythroid cells, plasmablasts and plasma linoleate diols [49, 50]. Thus, identifying features and differences amongst patients with varying severities of COVID-19 based on all omics data may prove more effective in understanding the potential therapeutic targets to modulate and assist in developing of predictive models of disease severity, resulting in patient better outcomes [18].

#### To investigate comorbidity in COVID-19

Hypertension, cardiovascular diseases (CVDs), diabetes mellitus chronic kidney diseases (CKDs), cancers and chronic obstructive pulmonary disease (COPD) are common comorbidities or pre-existing conditions in COVID-19 associated with worse outcomes [21, 51–53]. The risk of mortality in COVID-19 increases with age, gender and comorbidities, whilst age, male gender, shortness of breath, cerebrovascular disease and COPD were mortality-associated factors [16]. A multi-country study revealed that survival time from symptom onset was significantly shorter in elderly versus young patients, males versus females and patients with versus patients without comorbidities [16]. Multiomics studies contributed to identifying the pathway crosstalk between COVID-19 and comorbidities, helping to clarify these shared pathways and to identify gene-based targets for COVID-19 [21]. An integrated omics approach identified common comorbidities associated with COVID-19 including multiple systematic diseases or disorders, such as immune, pulmonary, cardiovascular, metabolic, liver, kidney and other systems [46]. Similarly, a high-throughput experimental data and protein interactome study identified the hub proteins in COVID-19, indicating the likelihood of symptoms similar to other lung diseases, such as asthma, COPD and pneumonia [41]. Meanwhile, a network medicine approach indicated that COVID-19 shared an intermediate inflammatory molecular profile with asthma [54]. Multiomics integration studies further identified the pathobiology, the viral modulated functional hubs of COVID-19 and pathway crosstalk between COVID-19 and other diseases, specifically considering pre-existing comorbid conditions [21, 41, 54]. Such findings demonstrated that COVID-19 shared pathobiology with inflammatory bowel disease [54], whilst the protein hubs in viral replication were correlated with hypertension, CVD and diabetes [41], and pathway crosstalk was identified between COVID-19 and diabetes, hypertension, CVDs and chronic kidney disease [21]. In addition, a pathway crosstalk was identified between COVID-19 and cancer [21], whilst the immune and genomic results demonstrated that a low triiodothyronine (T3) syndrome could coexist [55]. These multiomics-based results describe various risk factors in individuals, improving current gaps in personalized approaches to COVID-19.

#### To predict disease progression and outcomes in COVID-19

Multiomics integration data could be used to determine a biosignature to identify COVID-19 progression, prognostics and predicting outcomes amongst heterogeneous patients and guide therapeutic interventions. For example, multiomics profiles revealed that features of the stages of seroconversion could be used to stratify COVID-19 patients and correlate with different pathophysiological states, involving multiple underlying pathophysiological processes such as hyperinflammation and thrombotic microangiopathy [22]. Low antibody titers correlated with hyperactive T-cells, and the depletion of neutrophils, lymphocytes as well as platelets. Upon seroconversion, these biosignatures decrease or fully reverse, leading to an increase in B-cell subsets [22]. Meanwhile, D-dimer and hypoalbuminemia increased upon seroconversion microangiopathy [22]. Moreover, a multiomics study by Chen et al. [1] using transcriptomic, proteomic and metabolomic profiles provided the molecular features of the pathophysiology in disease progression and clinical outcomes, allowing for the construction of a COVID-19 prognostic classification model. Integrating these plasma omics profiles with multiple datasets as well as the features of expressed genes, proteins, exRNAs and biochemical parameters as potential biomarkers can help predict varying prognoses in COVID-19 patients and stratify those patients into groups [1]. Furthermore, novel blood biomarkers identified through a multiomics integration model revealed a clear distinction between a good and poor prognosis in COVID-19 [1]. For instance, integrating the clinical and proteomics datasets, increased blood clotting factor levels and a decline in the expression of the coagulation factor XIII A chain (F13A1) marker correlated with a poor prognosis in COVID-19 patients [1]. Meanwhile, megakaryocyte and erythroid cell derived co-expression modules derived from multiomics data were identified as predictors of fatal outcomes in COVID-19 [49]. Furthermore, a multiomics transition study demonstrated fluctuations in red blood cell (RBC) and hemoglobin (Hb) levels between survivors and non-survivors [36]. A network analysis revealed a late juncture in fatal COVID-19 based on the simultaneous assessment of proteins, transcriptomes and T-cell receptor sequences, whereby distinct circulating protein trajectories appeared useful in predicting recovery versus fatal outcomes [56]. These findings refine our comprehensive understanding of the pathophysiology and clinical progression of COVID-19, and ultimately could be used to improve COVID-19 interventions and therapies.

### Accelerated development of drugs, prevention tools and therapies for COVID-19

Despite multiple vaccines being widely and globally deployed, the pandemic continues to progress, and effective treatment remains largely unavailable. One of the primary purposes of understanding the characteristics of COVID-19 through multiomics integration data is to accelerate the development of drugs, vaccines, prevention tools and therapies for COVID-19. Multiomics data suggest that a high-frequency amino acid variation (AAV) in mutations could be considered for the novel design of a vaccine [32], whilst the clinical impact of deletions in ORF8 could be used to develop treatments and vaccines for COVID-19 [33]. Multiomics data with multiple source integration from patients could drive multiplex drug repurposing and offer rapid mapping and drug prioritization for COVID-19 [57]. Specifically, multiomics integrative analyses with a multiplex drug repurposing (DR) approach yielded a highly informed shortlist of drug candidates against COVID-19 and its causative virus, including drugs aiming to reverse COVID-19-induced perturbations (e.g. immunomodulatory and anti-inflammatory drugs) and compounds with a direct antiviral activity [57]. For instance, Src tyrosine kinase inhibitors hold a promising potential against COVID-19 [57]. Integrated interatomic, proteomic, transcriptomic and bibliomic profiles showed several prophylaxis agents (e.g. curcumin, vitamin D and melatonin) and candidate drugs (e.g. betamethasone and cyclosporin A) against COVID-19 [38]. A multimodal data harmonization approach identified a list of 84 drug target candidates and 7 high-confidence targets as potential starting points for drug therapy and development in COVID-19 [58], and also presented the potential candidate in phytocompounds and Chinese medicine [59, 60]. Meanwhile, multiomics and systems pharmacological findings revealed 154 compounds that targeted 13 immune genes involved in diverse signaling pathways, suggesting novel potential targets for COVID-19 treatment and prevention [60]. Thus, multivariate approaches to harmonize multiomics data demonstrated advantages in developing COVID-19 prevention and treatment methods, an approach that effectively worked in other diseases.

### Post-acute COVID-19 syndrome (PACS) characteristics

Mounting evidence suggests that symptoms may last for months after recovery from the initial COVID-19 infection [9, 61]. Whilst definitions vary with symptoms persisting for more than 4 to 12 weeks, a condition termed post-acute COVID syndrome (PACS) [23] or in some contexts simply long COVID has been defined [9, 62]. Whereas this group also includes patients with lingering symptoms after an initially acute severe disease which required hospitalization, the majority of this group consists of patients that initially experienced mild disease with little to no symptoms [25]. The latter group typically consists of patients who are young, previously healthy, and mentally and physically strong [63, 64]. In addition, the female gender appears to be twice as likely to develop long COVID as males [24]. The most common long-term sequelae symptoms include fatigue, dyspnea, chest pain, recurring fever, neurological complaints, olfactory dysfunctions, tachycardia, intestinal disorders and skin manifestations, and possibly unrelated to the severity of the initial acute infection [9, 24, 62–64]. In the Coverscan study [65], damage to at least one organ was observed in 70% of patients, with multiple organ damage appearing in 25% of patients with long COVID. Furthermore, long COVID does not appear to consist of prolonged recovery from the initial infection, but rather a cyclic condition with fatigue and a recurring fever exacerbated after activity. The upregulated omics profiles in COVID-19 might remain switched on, due to viral RNA remaining active in survivors, suggesting that a corresponding non-communicable disease may develop and lead to long-term consequences [46], whilst the long-term symptoms may change in nature over time [62]. A comprehensive understanding of the characteristics in post-COVID remains unmet, whereas very limited multiomics studies in this field have been reported. For instance, Barh et al. [46] used a multiomics approach to predict possible long-term complications of COVID-19, achieving high accuracy of over 90%. A multiomics-based bioinformatics approach identified the most common conditions accompanying COVID-19, noting that these play a potential role in the long-term consequences for COVID-19 survivors [46]. Hence, multiomics datasets may truly predict the growing effect of the disease beyond hospitalization and mortality, and accurately target care for survivors through the development of preventive and effective treatments for COVID-19 [9].

## Multiomics integration approaches

**Figure 2:**
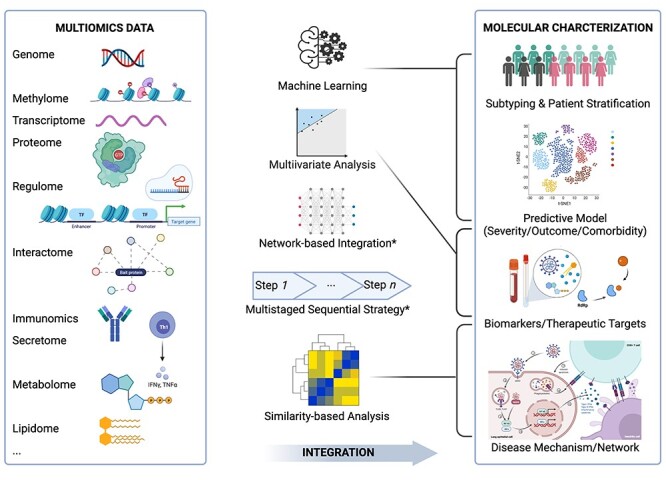
Summary of five categories of multiomics integration strategies and their application in the molecular characterization of COVID-19. Commonly used multiomics data (left box) are integrated through five categories of integration approaches (middle) to investigate four major applications in the molecular characterization of COVID-19 (right box). The grey lines from the middle to the right represent the major applications of approaches for specific purposes. Both network-based and multistaged strategies have been performed for all four applications. Created using BioRender.com.

The development of multiomics integration bioinformatics approaches has provided an opportunity to comprehensively understand a complex disease involving multiple molecular-level changes and their interactions, representing an important direction in systems and precision medicine. In previous reviews, these approaches could be grouped based on their purpose: disease subtyping and patient stratification; diagnostic and prognostic predictive model construction; and driving insight into disease biology through the identification of biomarkers, pathways and disease networks [30]. Such approaches could also be distinguished based on algorithms, such as multivariate, machine learning, network, correlation or similarity and a fusion of approaches using either multistaged (linear or sequential integration) or meta-dimensional (or simultaneous integration) integrative strategies. (Further details and a comparison of these latter approaches can be found in reviews presented elsewhere [30, 66, 67]). Given the exceptional heterogeneity and diversity of multiomics datasets as well as the widespread availability of external databases, multiomics integration processes are quite project-oriented and specific. Here, we summarize several commonly used approaches in the multiomics molecular characterization of COVID-19 (see Figure 2).

### Multivariate analyses

Multivariate analyses such as partial least squares (PLS) and its extension multi-block PLS are supervised multivariate analyses seeking predictable features and attempting to build predictive models for both the severity and outcomes of COVID-19. Liu et al. [56] built a logistic PLS model based on changing protein expressions in a longitudinal cohort to predict fatal outcomes in COVID-19. Similarly, Chen et al. performed a supervised PLS on each single omics dataset and multiblock PLS (DIABLO) to integrate the multiomics dataset, including proteome, transcriptomes, translatome, lipidome and metabolome data. They revealed a harmonized multimodal signature that can further assist in the discovery of drug targets [58]. In addition, mixOmics is a commonly used tool for multiomics integration, which also includes additional multivariate methods for future applications [68]. One advantage to multivariate analysis is its simultaneous ability to select features and generate predictable models. However, it carries a limited predictive power when the data are complex, such as with nonlinear relationships.

### Machine learning in predictive models

Machine learning approaches have a higher potential to construct efficient predictive models for complex and ambiguous data. Specifically, such approaches could be used in unsupervised COVID-19 subtyping and patient stratification, and in constructing supervised diagnostic and prognostic predictive models. For instance, Overmyer et al. [18] constructed an ExtraTrees classifier based on combining four omics datasets of metabolites, lipids, proteins and transcripts to predict disease severity. In a study by Song et al. [69], they first selected significantly differentiated lipids and polar metabolites comparing healthy and COVID-19 patients, and then constructed a predictive logistic regression model for COVID-19 with high accuracy (AUC = 0.975). Similarly, Chen et al. [1] selected variables and trained prognostic models using multiple machine learning algorithms, including the nearest mean classification (NMC), k-nearest neighbors (KNNs), support vector machine (SVM) and random forest (RF), through the integration of clinical measurements, exRNA-seq, mRNA-seq and proteomics datasets. Relatedly, Shen et al. [70] built an RF model based on proteomic and metabolomic data from 18 non-severe and 13 severe patients, further prioritizing 22 proteins and 7 metabolites using the mean decrease in accuracy. Generally, machine-learning approaches have the advantage of high accuracy, flexible requirements and hypotheses related to data distribution, and high compatibility with data types. Nevertheless, such approaches require a greater effort to avoid overfitting, a larger sample size, and further downstream explanations to link the predictable features or models with biological insights.

### Network-based multiomics integration to understand COVID-19 infection and the immune response

Network-based integration approaches have been increasingly applied in multiomics COVID-19 research given that such approaches match the nature of multilevel, complex and crosstalk interaction networks involved in SARS-CoV-2 infection and COVID-19 disease progression. Network approaches could illustrate and explain interactions amongst multilevel molecules, including DNA, RNA, protein, metabolites and their interactomes, as well as cellular interactions and immune responses across all processes from SARS-CoV-2 exposure to COVID-19 disease and post-COVID syndrome. Terracciano et al. [71] utilized a multilevel approach to mapping the SARS-CoV-2–host protein–protein interactome using affinity purification mass spectrometry with a graphical network representation combined with the simultaneous analysis of the host transcriptome, proteome, ubiquitinome and phospho-proteome following viral infections to discover host-directed anti-SARS-CoV-2 therapeutics. Similarly, Zhou et al. [26] incorporated SARS-CoV-2 virus–host protein–protein interactions, transcriptomics and proteomics into the human interactome to reveal the underlying pathogenesis. They further investigated COVID-19-associated disease manifestations by evaluating the network-based relationships of 64 diseases across enriched function categories and drug repurposing for COVID-19 [54]. In addition, Stukalov et al. [40] integrated the interactomes of both SARS-CoV-2 and SARS-CoV, examining their influence on the transcriptome, proteome, ubiquitinome and phosphoproteome of a lung-derived human cell line to identify host permutations in multilevel proteomics through a network diffusion approach. Understanding the immune response and system changes represents an important step to gaining insight into COVID-19, and identifying predictive and therapeutic signatures and targets. Additionally, Liu et al. [56] performed conditional independence network analysis to create a severity network through the identification of direct and indirect associations between enriched differentially expressed gene sets and a disease severity metric using a Lasso regression model. The development of single-cell omics technology allows for investigating the microenvironment and changes to the immune cell and their communication. In doing so, the B-cell receptor (BCR) and T-cell receptor (TCR) represent the most interesting and useful signatures for B-cell and T-cell responses and clonotypes. For example, Stephenson et al. [47] constructed a single-cell BCR clonotype network using adjacency matrices computed from the pairwise Levenshtein distance of the full amino acid sequence alignment for BCRs contained in every pair of cells from the peripheral blood mononuclear cells (PBMCs) within each COVID-19 severity cohort. Based on the clonotype network, Stephenson et al. [47] discovered some evidence of class switching within symptomatic COVID-19 groups, but not in asymptomatic or healthy individuals, whilst also observing some difference between men and women. Similarly, Tomazou et al. [57] devised a network-based integration of multiomics data, combining proteomics and metabolomics, transcriptomics, genomics and the pathogen–host network from COVID-19 patients and a drug repurposing cell line, attempting to prioritize the most important genes related to COVID-19 (gene–disease association ranking), subsequently reranking the identified candidate drugs. Whilst multilevel molecular and cellular networks have been investigated, there is enormous potential for network-based multiomics molecular research in COVID-19. Alongside the molecular network, a network-based patient similarity network has proven efficient in identifying disease subtypes and further predicting and stratifying patients in multiple cancers and chronic obstructive pulmonary disease. This line of inquiry represents a candidate for future research in COVID-19 patient diagnosis and outcome stratification.

### Multistaged sequential integration to identify disease-related biomarkers, pathways, or networks

Multistaged sequential approaches are typically used to integrate parallel analyses from each single-omics or layer of data. One method involves identifying differentially expressed signatures or genes from each single-omics platform, then merging these lists based on their direct overlap or further enriched downstream functions or pathways. For example, Chen et al. [1] identified severity-related multiomics signatures and their enriched functions and pathways, and then, further associated potential multi-organ damage based on tissue-enhanced proteins and functions. Interactome (both virus–host and human interactome), regulome, bibliome, gene ontology and pathways are commonly used external databases, combined in multiomics research. The more heterogeneous and diverse the data type, the greater the number of requirements to the bioinformatics approaches. Barh et al. [38] developed a strategy including gene set enrichment, lung-specific protein–drug network and candidate gene analysis to identify SARS-CoV-2 infection biology and candidate drugs to counter COVID-19 using interactome, bibliome, longitudinal proteome and transcriptome data. A multistaged method could flexibly integrate several analytical modules based on a specific research question and require cautious efforts to reproduce results.

### Similarity and correlation-based therapeutic targets and drug selection

The similarity between molecular changes and drug responses from a diseasome view relies on the basic premise of similarity or correlation-based analysis in COVID-19. For instance, Overmyer et al. [18] performed a cross-ome correlation analysis, correlating plasma proteins, metabolites and lipids omics data, identifying clusters further associated with COVID-19 patient status. Similarly, Song et al. [69] performed a differential correlation analysis of plasma lipids in mild COVID-19 cases relative to healthy controls, constructing a correlation network in mild COVID-19 cases. Su et al. [37] constructed a COVID-19 severity-dependent cross-omic interaction network, which included clinical measurements, plasma metabolomic and proteomic data. They further resolved an orchestrated response gene module that correlated with clinical features by integrating both high-dimensional bulk and single-cell multiomics profiles across cell types relying on a surprisal analysis [37]. This module could further characterize coordinated changes to cell types across COVID-19 patients. Similarity and correlation-based approaches could fast track links between COVID-19 and existing findings related to disease mechanisms, therapeutic targets and drugs from other similar diseases, thereby accelerating the development of therapeutics as well as prevention and diagnostic tools for both pre-COVID-19 medical conditions and post-COVID syndrome.

**Figure 3:**
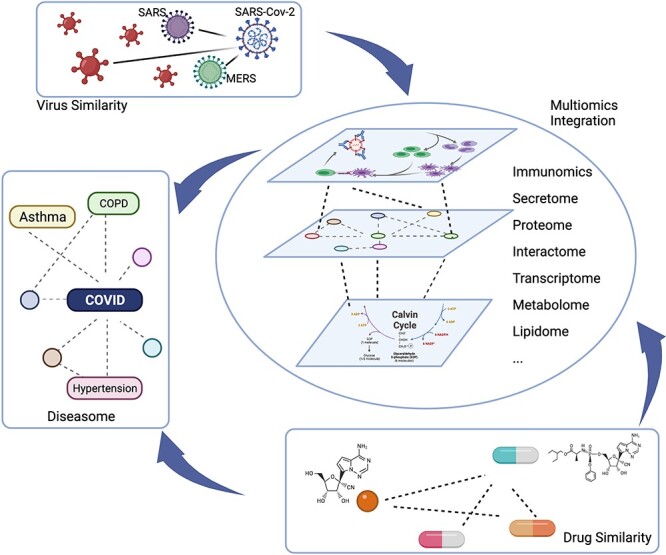
Illustration of the similarity-based assumptions for the transfer of previous knowledge to multiomics integration in COVID-19. Multiomics integration from immunomics, secretome, proteome, interactome, transcriptome, metabolome and lipidome amongst others could provide a systematic understanding of viral infection and COVID-19 disease progression and processes. SARS-CoV-2 is similar to SARS and MERS, as well as other viruses. Based on their similarities, the virus–host response and potential diagnostic and therapeutic targets derived from multiomics analyses could be transferred to and prioritized within COVID-19 research. Based on diseasome, given the similarity with known diseases, the candidate genes from diseases similar to COVID-19 could be analyzed and examined, particularly in relation to a predisposition to disease, comorbidities and in predicting long-COVID and characterizing patients. Drug similarities in terms of the chemical effects as well in the multiomics-level response could be used to prioritize candidate drugs and therapeutic targets. Created using BioRender.com.

## Trends and challenges in multiomics research on COVID-19

COVID-19 represents a challenging emergency both in terms of disease management and scientific research. Multiomics integration molecular characterization of COVID-19 patients will greatly assist preventive and personalized medicine efforts. In our review of 32 multiomics studies on COVID-19 published before July 2021 featuring at least two integrated omics datasets, we identified some trends and challenges in multiomics research on COVID-19.

### Systematic similarity-based association assumptions to improve understandings of COVID-19 mechanisms

Similarity-based assumptions, ranging from virus and disease phenotype to drug response correlation at the multiomics level, represent one fundamental hypothesis, which applies previous knowledge to COVID-19 research (see Figure 3). Briefly, multiomics-level reflections from immunomics, secretome, proteome, interactome, transcriptome, metabolome, lipidome and epigenome amongst others could provide a systematic understanding of viral infection and COVID-19 disease progression and processes. In addition, three levels of similarities could accelerate the transfer of previous knowledge to multiomics integration in COVID-19. SARS-CoV-2 is similar to SARS and MERS, as well as other viruses. Based on their similarities, the virus–host response and potential diagnostic and therapeutic targets from multiomics analyses could be transferred to and prioritized within COVID-19 research. Based on diseasome as the similarity of known diseases, the candidate genes from diseases similar to COVID-19 could be analyzed and examined, particularly in relation to a predisposition to disease, comorbidities, and in predicting long-COVID and characterizing patients. Drug similarities in terms of chemical effects as well in the multiomics-level response could be used to prioritize candidate drugs and therapeutic targets. Overall, the knowledge linking and transfer under the systematic similarity-based assumptions, encompassing virus and drug similarities, diseasome and multiomics molecular networks, may accelerate our understanding of COVID-19 mechanisms.

**Figure 4:**
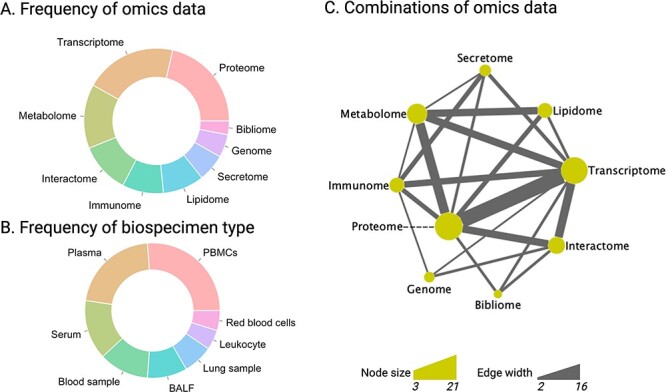
Summary of platform co-appearance in 32 multiomics studies of COVID-19. Pie charts of the prevalence of various omics platforms (A) and biospecimen types (B) that appeared in at least two publications, respectively. C) A network plot of the co-appearance of omics platforms, in which omics (node) appeared in at least two publications and omics pairs (edge) co-appeared in at least two publications. The size of the nodes corresponds to their appearance number in these publications (3 to 21). The width of the edge is related to the number of the co-appearances in these publications (2 to 16). Detailed information is available in Supplementary Tables 2–4. PBMCs, peripheral blood mononuclear cells; BALF, bronchoalveolar lavage fluid. Pie charts and network are created using R version 3.6.0. and Cytoscape version 3.8.2, respectively.

### Diversity and complexity of multiomics data and bioinformatics approaches pose a challenge to reproducibility

More than ten different kinds of omics or molecular signatures from over 15 biospecimen types have been used in the 32 multiomics studies discussed in this review (omics approach and biospecimen type used in each publication are detailed are summarized in Supplementary Tables 2 and 3). Nine of the 12 omics data types were used in at least 3 of 32 studies, including proteome, transcriptome, metabolome, interactome, immunome/signatures, lipidome, secretome/cytokine, genome and bibliome (Figure 4A). To highlight this data crossover, we constructed an omics co-appearance network, consisting of omics (node) that appeared in at least two publications and omics pairs (edge) that co-appeared in at least two publications. The size of nodes and the widths (weights) of edges correspond to the number of publications in which they were used (node) and co-appeared (edges), respectively. There are dense and complex combinations between these omics datasets. The seven most frequently used biospecimen types, used in at least two publications are visualized in Figure 4B, including PBMCs (corresponding to single-cell omics), plasma, serum, blood sample, BALF, lung sample, leukocyte and red blood cells (excluded unspecified/multiple recourse such as database and cell lines). Notably, epigenetics and epigomics play important roles in SARS-CoV-2 infection and COVID-19 pathogenesis, as discussed in Milad Shirvaliloo’s article, ‘Epigenomics of COVID-19 and the link between DNA methylation, histone modifications and SARS-CoV-2 infection’ [72]. In multiomics research, Zhao et al. [73] identified broad cellular effects from SARS-CoV-2 infection beyond adaptive immune cells through DNA methylome and transcriptome profiles. In addition, Shen et al. [70] revealed the crosstalk between perturbations taking place upon infection with SARS-CoV-2 and SARS-CoV at different levels, whereby multiomics were involved, including ubiquitinome and phosphoproteome. Multiple investigative opportunities on COVID-19 multiomics research exist in different multiomics combinations with diverse bioinformatics approaches, given the variety of clinical backgrounds.

One challenge when summarizing the multiomics data stems from the diversity and the ambiguous definition of omics data. For example, 32 cytokines expression levels are referred to as secretome in Su et al.’s study [37], but McReynolds et al. [50] referred to these as simply cytokines even though they analyzed 50 cytokines. Here, we grouped data into each omics type using an inclusive definition, whereby both Su et al. and McReynolds et al.’s studies are mapped into secretome/cytokine and both bulk and single-cell omics are attributed equal importance. It should be noted that the ambiguous definitions discussed above may have given rise to a certain level of inconclusiveness in our literature mining.

Another layer of challenges inherent in attempts to summarize such studies lies in the variety and complexity of bioinformatics approaches used. Multiomics integration is still an emerging and dynamic field, and standardized or generalized methods regarding the minimal number of combinations of omics, biospecimen types, clinical backgrounds, and other data modalities required to use the terminology, or approaches for mapping to PPI or pathways exist as of yet. The diversity and complexity in multiomics integration, on the one hand, provide greater possibilities to improve the statistical power and molecular depth of COVID-19 investigations, while on the other hand, the propensity for overfitting and difficulty in finding matching validation cohort may increase the difficulty in reproducing multiomics research, as well as accurately estimating systematic errors and bias overlap between data collected from different molecular levels and anatomical locations.

The availability of high-throughput multiomics datasets both in bulk and at the single-cell level, in combination with the availability of relatively comprehensive databases for protein interaction, functional annotation and molecular pathways offer vast opportunities for mechanistic interpretations of multiomics integration networks. Conversely, heterogeneous and diverse data types with complex internal relationships give rise to challenges to the computational approaches. Machine learning approaches along with more recently developed multiomics integration approaches such as Similarity Network Fusion [74] and iCluster [75] have proven highly efficient, but with a high risk of overfitting if the study design and cohort size is inappropriate for the chosen method. Another challenge lays in the gaps between the availability of powerful statistical methods based on thousands of molecular features, and a lack of matching clinical and biological explanations.

### Single-cell omics analyses in COVID-19

Single-cell omics provides an unprecedented potential for exploring biological systems also when sample sizes are scarce, and represents an emerging trend in COVID-19 research as well as in many other areas [76]. To name a few, Su et al. [37] performed single-cell multiomics analyses of PBMCs using whole transcriptome, surface proteins, secreted proteins and TCR and BCR gene sequence data, and thereby identified a sharp disease-state shift between mild and moderate COVID-19, suggesting that moderate COVID-19 may provide the most effective setting for therapeutic intervention [37]. Stephenson et al. performed single-cell transcriptome investigations of the immune response in COVID-19′ of 130 patients with varying severities of COVID-19, highlighting the comprehensive landscape of a coordinated immune response contributing to COVID-19 pathogenesis [47]. In a longitudinal multiomics COVID-19 study, the authors employed parallel scRNA-seq, single-cell BCR profiling, bulk mRNA sequencing (RNA-seq), BCR amplicon sequencing and multicolour flow cytometry from baseline to follow-up [49]. Through integrations of single-cell- and bulk sample data, their analysis identified a few hallmarks of severe COVID-19, such as megakaryocytes, erythroid cells and plasmablasts [49]. A more in-depth analysis on the subject on single-cell analyses in COVID-19 is provided in a recent review by Huo et al.’s review [77]. The integration of bulk- and single-cell multiomics also pose a new set of challenge for bioinformatics algorithms.

### The importance of cohort design in multiomics investigations

In this review, we discuss the current status of and potential for multiomics integration in COVID-19 research. The investigations performed to date primarily focused on predicting SARS-CoV-2 infection and COVID-19 severity. However, in order to harness the full potential of multiomics investigations, great care must be places on the study- and cohort design. We have previously shown that multiomics integration can increase the statistical power to classify small sub-groups of patients greatly, reducing the required group size to reach 95% power from *n* = 30 for single omics to *n* = 6 for 5–7-tuple omics integration. However, this level of statistical power was achieved in a rather homogeneous cohort (the Karolinska COSMIC cohort) resulting from very strict exclusion criteria in terms of co-morbidities, treatments, etc. [20]. In contrast, many of the COVID-19 studies discussed in this review were limited to 10–50 subjects, with 3 or fewer omics platforms included. In order to reach the full statistical potential in COVID-19 related multiomics integration studies, cohorts must either be designed strictly with specific sub-groups of patients and symptoms in mind, ideally collecting samples not only systemically but also at the site of injury, or with very large numbers. With these factors met, future breakthroughs may include identifying preventive and therapeutic targets, early diagnostic tools specifically for asymptomatic subjects and high-risk populations with medical conditions, effective predictive models of critical and severe outcomes, and the selection of drugs as well as further understanding comorbid conditions and the long-term complications and consequences of COVID-19.

### Development of user-friendly tools and database

Many multiomics datasets are available for further analysis, including several web-based dataset summaries (https://opendata.ncats.nih.gov/covid19/omics), as well as analytical and visualization tools, which are friendly for non-bioinformaticians. These tools help to quickly apply results and make available original data to the clinical and research communities. Given the diversity and complexity of multiomics, we found many studies associated with several databases or resources, along with varying depths of procession. Well-processed data in an integrated, standardized format will benefit the re-analysis and re-integration of data. Thus, we developed an online Shiny App termed ‘Collection of multiomics datasets in COVID-19’ (available via https://zhougroup.shinyapps.io/moCOVID/) to aid researchers and clinicians in searching COVID-related multiomics investigations (Table 1 and Supplementary Table 1), as well as a corresponding downloadable database and online tools.

## Conclusions

Like many other complex diseases, COVID-19 is an umbrella diagnosis with patient outcomes ranging from asymptomatic, to acute respiratory distress syndromes and cytokine storms requiring hospitalization and ICU treatment, to the debilitating long term symptoms of fatigue, vagal dysfunction, brain fog, postural orthostatic tachycardia syndrome (POTS), etc., experienced by patients with PACS. Identification of the molecular characteristics of this broad range of COVID-19 patients, powered by multiomics integration studies could offer a tremendous potential to unravel SARS-CoV-2 pathobiology, disease progression and resolution, predicting outcomes, prevention and therapy. Many lessons learned from multiomics integration in like various cancer types and respiratory disease can potentially transfer to COVID-19 research [20, 30, 66, 67]. New multiomics integration approaches, including multilevel virus–host interactions, handling of longitudinal data and dynamic models is needed. User-friendly, open access software programs and visualization tools are also desirable to increase accessibility of integration strategies the to a broader researcher base, beyond highly trained bioinformaticians, to facilitate efficient implementation of these results to clinical and biological research.

**Key Points**

- An increased and comprehensive understanding of COVID-19 characteristics is crucial to guiding the pandemic response.
- Identification of the molecular characteristics of COVID-19 patients from multiomics integration studies offers tremendous potential to unravel the puzzles related to SARS-CoV-2 pathobiology and COVID-19 progression.
- Multiomics integration-based molecular characterizations of COVID-19 could answer multiple important questions, specifically those related to disease severity, outcome prediction, prevention and therapy.
- To date, more than ten types of omics data and five categories of multiomics integration approaches have been applied to COVID-19 research, with further research potential and challenges in the biological and bioinformatics areas.

## Supplementary Material

BIB-21-1083_R1_suppl_table1_1110_bbab485

BIB-21-1083_R1_table2-4_1004_bbab485

## Notes

**Chuan-Xing Li, PhD**, is a researcher at Karolinska Institutet (Sweden) and the First Hospital of Lanzhou University (China). Her research interest lies in computational precision medicine and multiomics integration.

**Jing Gao, PhD, MD**, is a researcher at the Karolinska Institutet (Sweden), the University of Helsinki and Helsinki University Hospital (Finland), and the First School of Clinical Medicine at Lanzhou University (China). Her research interests include respiratory medicine and bioinformatics.

**Zicheng Zhang** is a graduate student in the School of Biomedical Engineering, Wenzhou Medical University (China). His research interests include bioinformatics and immunoinformatics.

**Lu Chen** is a graduate student in the School of Biomedical Engineering at Wenzhou Medical University (China). Her research interests include bioinformatics and disease systems biology.

**Xun Li, PhD, MD**, is a professor at the Department of General Surgery and Key Laboratory of Biotherapy and Regenerative Medicine of Gansu Province at the First Hospital of Lanzhou University (China). His research interests extend across multiple disease fields, including COVID-19.

**Meng Zhou** is an associate professor in the School of Biomedical Engineering at Wenzhou Medical University (China). His research interests include bioinformatics, computational precision medicine and immuno-oncology.

**Åsa M. Wheelock, PhD**, is an associate professor at the Respiratory Medicine Unit, Department of Medicine and the Centre for Molecular Medicine at the Karolinska Institutet, Stockholm, Sweden. Her research interests involve molecular sub-phenotyping of heterogeneous diagnoses of obstructive lung disease, such as COPD, asthma and as of recent also post-acute COVID syndrome (PACS) using multiomics integration and systems medicine approaches.
