# Supplementary material for: Multiomics integration-based molecular characterizations of COVID-19
Source: Brief Bioinform. 2021 Dec 2;23(1):bbab485. doi: 10.1093/bib/bbab485 (PMC8769889; doi:10.1093/bib/bbab485)
Supplement: BIB-21-1083_R1_suppl_table1_1110_bbab485 [file bib-21-1083_r1_suppl_table1_1110_bbab485.docx]

**Supplementary Table 1.** Summary and descriptions of COVID-19 multiomics publications including purpose and summary of results and number of samples or cell lines

| **Authors** | **Title** | **Subjects** | **Publication Date** | **Longitudinal** | **Number of Omics** | **Purpose** | **Results** | **Data download** |
| --- | --- | --- | --- | --- | --- | --- | --- | --- |
| Su, Y., et al. [37] | Multi-Omics Resolves a Sharp Disease-State Shift between Mild and Moderate COVID-19 | 139 COVID-19 patients (110 mild, 102 moderate, and 52 severe), and 258 healthy | 10/12/2020 | yes, 2 time points | 5 | 1. To identify a major shift between mild and moderate disease 2. To understand the coupling between elevated inflammation signals, plasma metabolite composition, and immune cell dysfunction in COVID-19 | 1. Analysis of serial blood from 139 COVID-19 patients reveals immune coordination 2. A major immunological shift observed between mild and moderate infection 3. Moderate and severe cases exhibit inflammation and a sharp drop in blood nutrients 4. Novel immune cell subsets emerge in moderate cases and increase with severity | Array Express under the accession number: E-MTAB-9357 and supplementary data at http://dx.doi. org/10.17632/tzydswhhb5.5. |
| Overmyer, K. A., et al. [18] | Large-scale Multi-omic Analysis of COVID-19 Severity | 102 COVID-19-positive patients and 26 COVID-19-negative patients | 20/01/2021 | no | 4 | To identify correlations between molecular factors and clinical data or patient outcomes | 1. Found 219 biomolecules strongly associated with COVID-19 status and severity 2. Observed pronounced dysregulation of lipid transport and neutrophil degranulation | The measurements and patient data are located at https://covid-omics.app; RNA-seq data is available at GEO(accession GSE157103). |
| Chen, Y. M., et al. [1] | Blood molecular markers associated with COVID-19 immunopathology and multi-organ damage | 50 mild COVID-19 patients, 16 severe COVID-19 patients, and 17 healthy volunteers | 15/12/2020 | 65 patients with more than one sampling time point | 3 | To understand host responses to COVID-19 pathophysiology and identify blood molecular markers associated with COVID-19 immunopathology and multi-organ damage | 1. A large number of expressed genes, proteins, metabolites, and extracellular RNAs (exRNAs) exhibit strong associations with various clinical parameters  2. Multiple sets of tissue-specific proteins and exRNAs varied significantly in both mild and severe patients suggesting a potential impact on tissue 3. Chronic activation of neutrophils, IFN-I signalling, and a high level of inflammatory cytokines were observed in patients with severe disease progression 4. COVID-19-infected patients experiencing milder disease symptoms showed robust T-cell responses  5. Identified genes, proteins, and exRNAs as potential biomarkers that might assist in predicting the prognosis of SARS-CoV-2 infection | RNA-Seq and exRNA-Seq Data: NODE OEP000868 (http://www.biosino.org/node/project/detail/OEP000868). Raw mass spectrometry data: iProX IPX 0002186001 (https://www.iprox.org/page/subproject.html?id=IPX0002186001) |
| Thomas, T., et al. [14] | Evidence of Structural Protein Damage and Membrane Lipid Remodeling in Red Blood Cells from COVID-19 Patients | 29 COVID-19 patients and 23 healthy controls | 06/11/2020 | no | 3 | To identify proteomic and metabolomic shifts in red blood cells that contribute to the severe hypoxemia in COVID-19 patients | 1. A significant impact of SARS-CoV-2 infection appeared on the red blood cells (RBCs) structural membrane homeostasis at the protein and lipid levels 2. RBCs from COVID-19 patients may be less capable of responding to environmental variations in haemoglobin oxygen saturation or oxidant stress | Raw data have been uploaded to ProteomeXchange with the identifier no. PXD022013, accessible at the following link: https://massive.ucsd.edu/ProteoSAFe/dataset.jsp?task=02b4fa2ccaaa411988eb8059c6a45544. |
| Zhou, Y., et al. [54] | A network medicine approach to investigation and population-based validation of disease manifestations and drug repurposing for COVID-19 | Varies across literature and datasets | 06/11/2020 | no | 3 | To identify SARS-CoV-2 pathogenesis, disease manifestations, and COVID-19 therapies using network medicine methodologies along with clinical and multi-omics observations | 1. Shared pathobiology between COVID-19 and inflammatory bowel disease  2. COVID-19 shares an intermediate inflammatory molecular profile with asthma  3. Identified melatonin as a potential prevention tool and treatment of COVID-19 | GSE147507, GSE63142, GSE130499, and GSE134809. Statements of data and code availability are available at https://doi.org/10.1038/s41586-020-2332-7. PPI networks have also been uploaded to NDEx (https://public.ndexbio.org/#/network/43803262-6d69-11ea-bfdc-0ac135e8bacf) |
| Tomazou, M., et al. [57] | Multi-omics data integration and network-based analysis drives a multiplex drug repurposing approach to a shortlist of candidate drugs against COVID-19 | Varies across literature and datasets | 01/05/2021 | no | 5 | To identify potential drug targets for COVID-19 | 1. Dexamethasone, remdesivir, and Src tyrosine kinase inhibitors appear to be involved in multiple COVID-19 pathophysiological mechanisms  2. Specific immunomodulators and anti-inflammatory drugs like dactolisib and methotrexate and inhibitors of histone deacetylase like hydroquinone and vorinostat with potential beneficial effects in their mechanisms of action 3. This multiplex drug repurposing approach, developed and utilized herein specifically for SARS-CoV-2, could offer a rapid mapping and drug prioritization against any pathogen-related disease | Raw sequencing data have been deposited in the Genome Sequence Archive in BIG Data Centre (https://bigd.big.ac.cn/), Beijing Institute of Genomics (BIG), Chinese Academy of Sciences, under the accession number: CRA002390. GSE147507 |
| Barh, D., et al. [38] | Multi-omics-based identification of SARS-CoV-2 infection biology and candidate drugs against COVID-19 | Varies across literature and datasets | 10/10/2020 | no | 4 | To present the biological events associated with SARS-CoV-2 infection and identify appropriate drug targets | 1. Viral process, mRNA splicing, cytokine and interferon signaling, and ubiquitin mediated proteolysis are important pathways in SARS-CoV-2 infection 2. SARS-CoV-2 infection shares pathways with Influenza A, Epstein-Barr virus, HTLV-I, Measles, and Hepatitis virus 3. Bacterial, parasitic, and protozoan infection pathways are shared by this virus 4. A total of 50 candidate drugs, including the prophylaxis agents and pathway specific inhibitors are identified against COVID-19 5. Betamethasone, Estrogen, Simvastatin, Hydrocortisone, Tositumomab, Cyclosporin A etc. are among the important drugs 6. Ozone, Nitric oxide, plasma components, and photosensitizer drugs are also identified as possible therapeutic candidates 7. Curcumin, Retinoic acids, Vitamin D, Arsenic, Copper, and Zinc may be the candidate prophylaxis agents | https://www.ebi.ac.uk/pride/archive/projects/PXD018117. https://public.ndexbio.org/#/network/43803262-6d69-11ea-bfdc-0ac135e8bacf |
| Zhao, Y., et al. [73] | Omics study reveals abnormal alterations of breastmilk proteins and metabolites in puerperant women with COVID-19 | 4 female COVID-19 patients and 2 healthy controls | 23/10/2020 | no | 3 | To determine whether breast milk is affected by COVID-19 | Breastfeeding or breast milk with a deficiency of immune-related components may not be beneficial to neonates in establishing an immune defence early in life | https://static-content.springer.com/esm/art%3A10.1038%2Fs41392-020-00362-w/MediaObjects/41392_2020_362_MOESM3_ESM.xlsx; https://static-content.springer.com/esm/art%3A10.1038%2Fs41392-020-00362-w/MediaObjects/41392_2020_362_MOESM7_ESM.xlsx; https://static-content.springer.com/esm/art%3A10.1038%2Fs41392-020-00362-w/MediaObjects/41392_2020_362_MOESM8_ESM.xlsx |
| Yang, R., et al. [59] | Chemical composition and pharmacological mechanism of Qingfei Paidu Decoction and Ma Xing Shi Gan Decoction against Coronavirus Disease 2019 (COVID-19): In silico and experimental study | 1 decoction preparation | 29/04/2020 | no | 2 | To evaluate the chemical composition and pharmacological mechanisms of the Qingfei Paidu Decoction as a Chinese medicine used for COVID-19 in China | The therapeutic effects of the Qingfei Paidu Decoction against COVID-19 may be attributed to the anti-inflammatory effects of the Ma Xing Shi Gan Decoction | Data for this project available from the corresponding author upon request. Supplementary materials: https://www.ncbi.nlm.nih.gov/pmc/articles/PMC7194979/ |
| Gupta, V., et al. [32] | Comparative Genomics and Integrated Network Approach Unveiled Undirected Phylogeny Patterns, Co-mutational Hotspots, Functional Crosstalk and Regulatory Interactions in SARS-CoV-2 | 245 SARS-CoV-2 genomes and varies across literature and datasets | 23/02/2021 | no | 2 | To understand the population-specific variations attributed to the high rate of infections in specific geographical regions | 1. Rigorous phylogenomic network analysis of complete SARS-CoV-2 genomes inferred five central clades named a (ancestral), b, c, d, and e (subtypes e1 and e2). Clade d and subclade e2 were found exclusively comprised of U.S. strains.  2. Host proteins, MYO5A, MYO5B, and MYO5C had maximum interaction with viral proteins (nucleocapsid [N], spike [S], and membrane [M] proteins). Blocking the internalization pathway by inhibiting MYO5 proteins which could be an effective target for COVID-19 treatment.  3. The functional annotations of the host-pathogen interaction (HPI) network were found to be closely associated with hypoxia and thrombotic conditions, confirming the vulnerability and severity of infection. 4. Screened CpG islands in Nsp1 and N conferring the ability of SARS-CoV-2 to enter and trigger zinc antiviral protein (ZAP) activity inside the host cell. 5. Compositional structural biology results suggested that mutations have a balance of opposing forces affecting pathogenicity suggesting that only a few mutations are effective at the translation level.  6. Novel HPI analysis and CpG predictions elucidate the proof of concept of hypoxia and thrombotic conditions in several patients. | [https://www.ncbi.nlm.nih.gov/labs/virus/vssi/#/virus?SeqType_s= Nucleotide%20seafood=%20market=%20pneumonia=%20virus,=%20taxid:2697049=&VirusLineage_ss=Severe %20acute%20respiratory%20syndrome%20coronavirus%202%20(SARS-CoV-2),%20taxid:2697049](https://www.ncbi.nlm.nih.gov/labs/virus/vssi/#/virus?SeqType_s=Nucleotide%20seafood=%20market=%20pneumonia=%20virus,=%20taxid:2697049=&VirusLineage_ss=Severe%20acute%20respiratory%20syndrome%20coronavirus%202%20(SARS-CoV-2),%20taxid:2697049) |
| Shen, B., et al. [70] | Proteomic and Metabolomic Characterization of COVID-19 Patient Sera | 46 COVID-19 patients, and 53 control patients | 28/05/2020 | no | 2 | To predict disease severity from serum protein and metabolite levels, and to identify dysregulated pathways that can serve as potential therapeutic targets | 1. Performed proteomic and metabolomic profiling of sera from 46 COVID-19 and 53 control individuals 2. Trained a machine learning model using proteomic and metabolomic measurements and validated 3. Identified molecular changes in the sera of COVID-19 patients compared to other groups implicating dysregulation of macrophage, platelet degranulation, complement system pathways, and massive metabolic suppression 4. Revealed characteristic protein and metabolite changes in the sera of severe COVID-19 patients and could be contributed to the selection of potential blood biomarkers for severity evaluation | https://www.iprox.org/. Project ID: IPX0002106000 and IPX0002171000 |
| Song, J. W., et al. [69] | Omics-Driven Systems Interrogation of Metabolic Dysregulation in COVID-19 Pathogenesis | 26 healthy controls, 18 mild cases, 19 moderate cases, and 13 severe cases | 24/06/2020 | no | 2 | To identify metabolomic and lipidomic markers in patient sera distinguishing severe patients, mild patients, and healthy controls | Monosialodihexosyl ganglioside (GM3)–enriched exosomes may take part in pathological processes related to COVID-19 pathogenesis and presents the largest repository of the plasma lipidome and metabolome distinct for COVID-19 | http://dx.doi.org/10.17632/6z5zkzb3hv.3 |
| Young, B. E., et al. [33] | Effects of a major deletion in the SARS-CoV-2 genome on the severity of infection and the inflammatory response: an observational cohort study | 92 patients (wild-type SARS-CoV-2), 29 patients (the SARS-CoV-2 variant), and 10 patients (the wild-type and variant strains of SARS-CoV-2) | 18/08/2020 | yes, | 2 | To investigate the effect of SARS-CoV-2 variants with a 382-nucleotide deletion on the clinical features of infection | 1. The 382-nucleotide deletion variant of SARS-CoV-2 appears associated with a milder infection  2. The observed clinical effects of deletions in the open reading frame 8 (ORF8) could have implications for the development of treatment and vaccines | https://ars.els-cdn.com/content/image/1-s2.0-S0140673620317578-mmc1.pdf |
| Bruzzone, C., et al. [39] | SARS-CoV-2 infection dysregulates the metabolomic and lipidomic profiles of serum | 263 (training cohort) and 135 (validation cohort) symptomatic COVID-19 patients, and 280 persons selected before the coronavirus pandemic started | 05/10/2020 | no | 2 | To understand how SARS-CoV-2 affects metabolism | 1. Using Nuclear Magnetic Resonance (NMR) spectroscopy, we measured the metabolomic and lipidomic serum profile from training and validation cohorts of symptomatic patients hospitalised after positive PCR testing for SARS-CoV-2 infection  2. Established the profiles of 280 persons collected before the coronavirus pandemic began  3. The lipidomic analysis unravelled a pathogenic redistribution of the lipoprotein particle size and composition to increase the atherosclerotic risk  4. Metabolomic analysis revealed abnormally high levels of ketone bodies and 2-hydroxybutyric acid  5. Consistent with a model in which SARS-CoV-2 infection induces liver damage associated with dyslipidemia and oxidative stress | https://ars.els-cdn.com/content/image/1-s2.0-S2589004220308373-mmc1.pdf |
| Barberis, E., et al. [78] | Large-Scale Plasma Analysis Revealed New Mechanisms and Molecules Associated with the Host Response to SARS-CoV-2 | 19 critical patients, 84 non-critical patients, 32 COVID-19-negative patients with symptoms, and 26 healthy controls | 16/11/2020 | no | 2 | To characterise metabolomics and lipidomics profiles associated with a host immune response to COVID-19 to identify potential new therapeutic targets | 1. Several circulating lipids, such as phosphatidylcholine, phosphatidylcholine, and phosphatidylethanolamine, acted as potential biomarkers for COVID-19 2. Triglycerides and free fatty acids correlated well with the severity of disease  3. An untargeted analysis of noncritical COVID-19 patients identified a strong alteration of lipids 4. The severity of disease was characterised by the activation of gluconeogenesis and the metabolism of porphyrins, which play a crucial role in disease progression  5. Phospholipase A2 (PLA2) activity as a potential key factor in the pathogenesis of COVID-19 and a possible therapeutic target | Deposited to the EMBL-EBI MetaboLights database with the identifier MTBLS1866 |
| Islam, Abmmk K., et al. [35] | Transcriptome of nasopharyngeal samples from COVID-19 patients and a comparative analysis with other SARS-CoV-2 infection models reveal disparate host responses against SARS-CoV-2 | 4 nasopharyngeal samples, 2 lung biopsies from uninfected patients, 22 lung samples from deceased COVID-19 patients, 5 lung samples from deceased uninfected patients, and 2 cell lines | 07/01/2021 | no | 3 | 1. To profile the host transcriptome of COVID-19 patients from nasopharyngeal samples along with virus genomic features isolated from the respective host  2. To complete comparative analyses of differential host responses in various SARS-CoV-2 infection systems | 1. Unique and rare missense mutations in 3C-like protease observed in all of their reported isolates  2. Demonstrated that the host-induced responses are mediated by innate immunity, interferon, and cytokine stimulation  3. An upregulation of immune and cytokine signalling genes observed in the lungs  4. Lungs lacked the overexpression of ACE2 as suspected, although high ACE2 but low DPP4 expressions were observed in nasopharyngeal cells  5. Viral proteins, specifically the nonstructural protein-mediated overexpression of integrins in the lungs compared to nasopharyngeal samples, suggests a possible route of enhanced invasion  6. Comparatively high expression of transcription factors plays pivotal roles in lung injury | SARS‐CoV‐2 genomes are deposited at GISAID initiatives portal (https://www. gisaid.org/) with accessions‐ EPI_ISL_450340, EPI_ISL_450341, EPI_ISL_450342, EPI_ISL_450345.  Raw RNA‐seq data are deposited at NCBI‐GEO (https://www. ncbi.nlm.nih.gov/geo/) under the accession‐ GSM4667504, GSM4667505, GSM4667506, GSM4667507. |
| Chen, T., et al. [58] | A multi-modal data harmonisation approach for discovery of COVID-19 drug targets | Varies across literature and datasets | 24/05/2021 | no | 4 | To present a generic, reproducible, and flexible open-access data harmonisation framework that can be scaled to future multiomics analyses and applied it to a drug screening task in COVID-19 | 1. The two datasets were used for drug-target analysis, resulting in a list of 84 drug–target candidates  2. Computational docking and toxicity analyses revealed seven high-confidence targets (amsacrine, bosutinib, ceritinib, crizotinib, nintedanib, and sunitinib) as potential starting points for drug therapy and development | https://doi.org/10.5281/zenodo.4562010 |
| Barh, D., et al. [46] | A novel multi-omics-based highly accurate prediction of symptoms, comorbid conditions, and possible long-term complications of COVID-19 | Varies across literature and datasets | 08/03/2021 | no | 4 | To investigate comprehensive clinical pictures, comorbid conditions, and the long-term complications of COVID-19 | 1. Except for a dry cough and a loss of taste, all other COVID-19-associated mild and severe symptoms are enriched  2. Cardiovascular diseases (CVDs), and pulmonary, metabolic, musculoskeletal, neuropsychiatric, kidney, liver, and immune system disorders represent the most common comorbid conditions  3. Specific diseases along with several other non-communicable diseases represented the top candidates  4. Many cancers and congenital disorders associated with COVID-19 severity identified  5. Arthritis, gliomas, diabetes, psychiatric disorders, and CVDs with a bidirectional relationship with COVID-19 also identified as the most common conditions  6. Based on our accuracy (>90%), a long-term presence of SARS-CoV-2 RNA remains in humans | Data for this project is available from the corresponding author upon request. |
| Sciacchitano, S., et al. [55] | Gene signature and immune cell profiling by high-dimensional, single-cell analysis in COVID-19 patients, presenting Low T3 syndrome and coexistent hematological malignancies | 38 patients (low levels of FT3) and 24 patients (normal FT3) hospitalized during the first wave of SARS-CoV-2 outbreak | 01/04/2021 | no | 3 | To analyse the clinical relevance of low T3 syndrome in COVID-19 patients, in particular, amongst those with associated haematological malignancies | 1. Low FT3 serum levels associated with severe COVID-19  2. T3 involved in the immune response to COVID-19, and the coexistence of a haematological malignancy and new possible T3 target genes in such patients identified |  |
| Muthuramalingam, P., et al. [60] | Global multi-omics and systems pharmacological strategy unravel the multi-targeted therapeutic potential of natural bioactive molecules against COVID-19: An in silico approach | Public database or databases: ArrayExpress, PubChem, and WHO prescribed COVID-19 drugs list | 07/08/2020 | no | 3 | To unravel the multitargeted mechanisms of novel bioactives to combat COVID-19 using multiomics and systems pharmacological analyses | 1. Among 259 plant-derived compounds, 154 compounds targeting 13 COVID-19 immune genes involved in diverse signalling pathways 2. Pharmacological properties of these phytocompounds compared with COVID-19 drugs prescribed by WHO, and 25 novel phytocompounds found to be more efficient with higher bioactive scores | The human immuno-transcriptomic dataset of healthy controls and COVID-19 cases (E-MTAP-8871) was collected and retrieved from the ArrayExpress database (www.ebi.ac.uk/arrayexpress/) |
| Bernardes, J. P., et al. [49] | Longitudinal Multi-omics Analyses Identify Responses of Megakaryocytes, Erythroid Cells, and Plasmablasts as Hallmarks of Severe COVID-19 | 14 patients | 26/11/2020 | yes, 5 time points. | 4 | To provide a comprehensive, longitudinal view of cellular features in COVID-19 by using an integrated multiomics approach | 1. Severe COVID-19 characterised by an increase in proliferating, metabolically hyperactive plasmablasts  2. Expansion of interferon-activated circulating megakaryocytes and increased erythropoiesis with features of hypoxic signalling  3. Megakaryocyte and erythroid cell-derived co-expression modules indicative of a fatal disease outcome | 1. The bulk RNA-seq and BCR-seq data generated during this study available from the GEO database (GSE161777) 2. The Illumina EPIC Array data generated during this study available from the GEO database (GSE161678)  3. The raw single-cell RNA-seq data and single-cell BCR-seq data generated during this study available from EGA |
| Sun, C., et al. [36] | Longitudinal multi-omics transition associated with fatality in critically ill COVID-19 patients | 33 severe and critical COVID-19 patients without selected comorbidities and 114 critical COVID-19 patients | 15/03/2021 | yes | 4 | To perform a longitudinal investigation of the potential mechanisms affecting the prognosis of critically ill COVID-19 patients | 1. Frequent transcriptional phase transitions may contribute to outcomes in critically ill COVID-19 patients  2. Fluctuations in red blood cell and haemoglobin levels may associate with a poor prognosis  3. The biological function of melatonin suppressed in COVID-19 deaths | <https://www.ebi.ac.uk/biostudies/studies/S-EPMC7957447?xr=true> |
| Terracciano, R., et al. [71] | Mapping the SARS-CoV-2-Host Protein-Protein Interactome by Affinity Purification Mass Spectrometry and Proximity-Dependent Biotin Labeling: A Rational and Straightforward Route to Discover Host-Directed Anti-SARS-CoV-2 Therapeutics | Varies across literature and datasets | 07/01/2021 | no | 2 | To identify host-directed anti-SARS-CoV-2 therapeutics using protein-protein interactions analysis | 1. Generating protein-protein interaction maps may help to uncover cellular processes hijacked and/or altered by SARS-CoV-2, providing promising therapeutic targets  2. Drug repurposing might shorten the time and reduce the costs in comparison to the traditional drug discovery process | Data for this project is available from the corresponding author upon request. |
| Stukalov, A., et al. [40] | Multilevel proteomics reveals host perturbations by SARS-CoV-2 and SARS-CoV | The two viruses of SARS-CoV-2 and SARS-CoV | 12/04/2021 | no | 5 | 1. To project multiomics data onto the global network of cellular interactions revealing the crosstalk between perturbations taking place upon infection with SARS-CoV-2 and SARS-CoV at different levels 2. To identify of distinct and common molecular mechanisms of these closely related coronaviruses | 1. The TGF-β pathway specifically dysregulated by SARS-CoV-2 ORF8 and the autophagy specifically dysregulated by SARS-CoV-2 ORF3  2. The extensive dataset highlights many hotspots that could be targeted using existing drugs and may be employed to guide the rational design of virus- and host-directed therapies 3. Inhibitors of kinases and matrix metalloproteases with potent antiviral effects against SARS-CoV-2 | Raw sequencing data for this study have been deposited with the ENA at EMBL-EBI under accession number PRJEB38744.  The mass spectrometry proteomics data have been deposited to the ProteomeX change Consortium via the PRIDE83 partner repository with the dataset identifiers PXD022282, PXD020461 and PXD020222.  Protein interactions identified in this study have been submitted to the IMEx (https://www.imexconsortium.org) consortium through IntAct84 with the identifier IM-28109.  The data and analysis results are accessible online via the interactive web interface at https://covinet.innatelab.org. |
| Chen, X. J., et al. [79] | Novel insight from the first lung transplant of a COVID-19 patient | 36 samples were collected from the lungs | 20/11/2020 | no | 3 | To reveal detailed histopathological changes, virus distributions, immunological properties, and multiomic features caused by SARS-CoV-2 in the explanted lungs from the world's first successful lung transplantation of a COVID-19 patient | The significant neutrophil / CD3+ CD4- T-cell / macrophage activation leads to a cytokine storm and severe fibrosis in the lungs of a COVID-19 patient | Online supplement |
| McReynolds, C. B., et al. [50] | Plasma Linoleate Diols Are Potential Biomarkers for Severe COVID-19 Infections | Oxylipin analysis: 6 COVID-19 patients and 44 samples health control; cytokines: 75 samples from healthy volunteer | 01/04/2021 | no | 2 | To focus on oxylipin chemical mediators in COVID-19 disease | 1. Found increases in the regioisomeric leukotoxin diols in plasma samples of hospitalised patients suffering from severe pulmonary involvement 2. Incorporating diols in the plasma multiomics of patients possibly unravelling the COVID-19 pathological signature along with other lipid mediators and blood chemistry | https://datadryad.org/stash/dataset/doi:10.25338/B8M92X |
| Barh, D., et al. [21] | Predicting COVID-19-Comorbidity Pathway Crosstalk-Based Targets and Drugs: Towards Personalized COVID-19 Management | The same dataset as PMID: 33683246 | 17/05/2021 | no | 3 | To understand how comorbid conditions and COVID-19 interact at the molecular level, so that personalized management strategies can be adopted | 1. Identified the pathway crosstalk between COVID-19 and diabetes, hypertension, cardiovascular diseases (CVDs), chronic kidney diseases (CKDs), and cancers  2. Shared pathways and hub gene–based targets for COVID-19 and its associated specific and combination of comorbid conditions likely to lead towards the development of personalised management strategies | https://www.mdpi.com/article/10.3390/biomedicines9050556/s1 |
| Chen, Y., et al. [80]* | Proteomic Analysis Identifies Prolonged Disturbances in Pathways Related to Cholesterol Metabolism and Myocardium Function in the COVID-19 Recovery Stage | 10 patients from timeline study | 02/07/2021 | yes, disease onset and recovery stages | 1 | To investigate disease sequelae in patients recovering from COVID-19 and the mechanisms underlying these sequelae | The authors profiled the serum proteome of a cohort of COVID-19 patients in the disease onset and recovery stages. Based on the close integration of their proteomic analysis with clinical data, they propose that COVID-19 is associated with prolonged disorders in cholesterol metabolism and myocardium, even in the recovery stage. | iProX (integrated proteome resources) of ProteomeXchange with the accession code PXD023305. |
| Ahmed, S., et al. [41] | Regulatory Cross Talk Between SARS-CoV-2 Receptor Binding and Replication Machinery in the Human Host | Varies across literature and datasets | 30/06/2020 | no | 4 | 1. To describe the mechanism of SARS-CoV-2 in human lung hosts from the initial phase of receptor binding to the viral replication machinery  2. To test the functional role of the hubs derived from the interactome | 1. Most hubs proteins were differentially regulated in SARS-CoV-2 infection  2. The proteins in viral replication hubs were associated with cardiovascular disease, diabetes, and hypertension confirming the vulnerability and severity of infection among individuals at risk  3. The hub proteins were closely linked with other viral infections, including Middle East Respiratory Syndrome (MERS) and human coronaviruses (HCoVs), suggesting a similar infection pattern in SARS-CoV-2  4. Identified five hubs that interconnect both networks to show the preparation of the optimal environment in the host for the viral replication process upon receptor attachment  5. Seven potential miRNAs targeting the intermediate phase that connects the receptor and viral replication process, representing a better choice as a drug for SARS-CoV-2 | SARS-CoV2 host protein interaction dataset (https://doi.org/10.1101/2020.03.22.002386). RNA-seq dataset (GSE147507) |
| Galbraith, M. D., et al. [22] | Seroconversion stages COVID19 into distinct pathophysiological states | Clinical data from participation in the COVID Biobank of the University of Colorado Anschutz Medical Campusants | 16/03/2021 | no | 5 | To report the results of a cross-sectional multiomics analysis of hospitalised COVID-19 patients indicating that the seroconversion status associates with distinct underlying pathophysiological states | 1. Low antibody titers associate with hyperactive T-cells and NK cells, high levels of IFN alpha, gamma and lambda ligands, markers of systemic complement activation, and the depletion of lymphocytes, neutrophils, and platelets in COVID-19 2. Upon seroconversion, all of these processes were attenuated, demonstrating instead increases in B-cell subsets, emergency hematopoiesis, increased D-dimer, and hypoalbuminemia in COVID-19 3. The seroconversion status could be potentially used as a biosignature to stratify patients for therapeutic interventions and to inform the analysis of clinical trial results in heterogeneous patient populations in COVID-19 | https://flowrepository.org/id/RvFrSYioKeUdYHXdkTD9TQPAXt4PqdkB5eie82h11JgAGSCQIneLKpcKd81Nzgwq |
| Stephenson, E., et al. [47] | Single-cell multi-omics analysis of the immune response in COVID-19 | 130 patients with varying severities of COVID-19 | 20/04/2020 | no | 3 | 1. To perform single-cell transcriptome, surface proteome, and T- and B-lymphocyte antigen receptor analyses of patients with varying severities of COVID-19 2.To provide insights into the coordinated response to viral SARS-CoV-2 infections | 1. Identified the expansion of nonclassical monocytes expressing complementary transcripts  2. Early, uncommitted CD34+ hematopoietic stem and progenitor cells were primed towards megakaryopoiesis, accompanied by expanded megakaryocyte-committed progenitors and increased platelet activation  3. Clonally expanded CD8+ T-cells and an increased ratio of CD8+ effector T-cells to effector memory T-cells characterised severe disease, while circulating follicular helper T-cells accompanying mild disease  4. Observed a relative loss of IgA2 in symptomatic disease despite an overall expansion of plasmablasts and plasma cells | https://covid19cellatlas.org/ |
| Liu, C., et al. [56] | Time-resolved systems immunology reveals a late juncture linked to fatal COVID-19 | Hospitalised patients and 14 age- and gender-matched healthy controls; 33 hospitalised patients, and 38 critically ill patients who died or recovered | 10/02/2021 | yes, 4 time points | 4 | To longitudinally assess circulating proteins in single peripheral immune cells from COVID-19 patients to link immune response variation to disease severity and outcome over time | 1. Conditional-independence network analysis revealed primary correlates of disease severity 2. CD8+ T-cell activation apparent without signs of exhaustion  3. Although cellular inflammation was depressed in severe patients early after hospitalisation, it became elevated by days 17–23 following symptom onset, indicating a late wave of inflammatory responses, and circulating protein trajectories divergent between and predictive of recovery versus fatal outcomes | GEO database: GSE161918 |

Note: COVID-19, Coronavirus disease 2019; SARS‑CoV‑2, Severe acute respiratory syndrome coronavirus 2; exRNAs, extracellular RNAs; RBCs, Red blood cells; MYO, myosin; ∆382, the 382-nucleotide deletion; ORF8: the open reading frame 8; NMR spectroscopy, nuclear magnetic resonance spectroscopy; PLA2, phospholipase A2; ACE2, angiotensin-converting enzyme 2; DPP4,dipeptidyl-peptidase-4; CVDs, cardiovascular diseases; NCDs: non-communicable diseases; FT3: free triiodothyronine; TGF-β: transforming growth factor beta; CKDs, diabetes mellitus chronic kidney; sc: single-cell; MERS, Middle East respiratory syndrome; HCoV, human coronavirus. *: Multiple proteome data blocks from several longitudinal timepoints. The online searchable format shiny app can be found at ‘Collection of multiomics datasets in COVID-19’ (https://zhougroup.shinyapps.io/moCOVID/)
