# Supplementary material for: Multiomics integration-based molecular characterizations of COVID-19
Source: Brief Bioinform. 2021 Dec 2;23(1):bbab485. doi: 10.1093/bib/bbab485 (PMC8769889; doi:10.1093/bib/bbab485)
Supplement: BIB-21-1083_R1_table2-4_1004_bbab485 [file bib-21-1083_r1_table2-4_1004_bbab485.docx]

**Supplementary Table 2. Main omics and molecular signatures and the corresponding number of publications**

| Omics/Signatures | Number of publications |
| --- | --- |
| Proteome | 21 |
| Transcriptome | 20 |
| Metabolome | 14 |
| Interactome | 11 |
| Immunome/Signature | 9 |
| Lipidome | 9 |
| Secretome/Cytokine | 6 |
| Genome | 5 |
| Bibliome | 3 |

**Supplementary Table 3. Main biospecimen types and the corresponding number of publications**

| Biospecimen type | Number of publications |
| --- | --- |
| PBMCs | 11 |
| Plasma | 9 |
| Serum | 6 |
| Blood sample | 5 |
| BALF | 4 |
| Lung sample | 3 |
| Leukocyte | 2 |
| Red blood cells | 2 |

Note: PBMCs, peripheral blood mononuclear cells; BALF, bronchoalveolar lavage fluid.

**Supplementary Table 4. Omics pairs and the corresponding number of publications**

| Omics 1 | Omics 2 | Number of Publications |
| --- | --- | --- |
| Proteome | Transcriptome | 16 |
| Proteome | Metabolome | 10 |
| Transcriptome | Interactome | 9 |
| Proteome | Interactome | 8 |
| Metabolome | Lipidome | 8 |
| Metabolome | Transcriptome | 8 |
| Transcriptome | Immunome/Signature | 7 |
| Proteome | Lipidome | 5 |
| Proteome | Immunome/Signature | 5 |
| Secretome/Cytokine | Immunome/Signature | 5 |
| Transcriptome | Secretome/Cytokine | 4 |
| Proteome | Secretome/Cytokine | 3 |
| Proteome | Bibliome | 3 |
| Lipidome | Transcriptome | 3 |
| Transcriptome | Bibliome | 3 |
| Interactome | Genome | 3 |
| Interactome | Bibliome | 3 |
| Metabolome | Secretome/Cytokine | 2 |
| Metabolome | Immunome/Signature | 2 |
| Transcriptome | Genome | 2 |
| Genome | Immunome/Signature | 2 |
